# Soft tissue calcifications in chronic kidney disease—beyond the vasculature

**DOI:** 10.1007/s00424-025-03098-0

**Published:** 2025-06-05

**Authors:** Abul Fajol, Christian Faul

**Affiliations:** Section of Mineral Metabolism, Division of Nephrology, Department of Medicine, Heersink School of Medicine, The University of Alabama at Birmingham, Birmingham, AL USA

**Keywords:** Calcification, Chronic kidney disease, Hyperphosphatemia, Phosphate

## Abstract

Inappropriate mineralization of soft tissues, also called ectopic calcification, is a well-known pathology in chronic kidney disease (CKD) that is associated with increases in systemic phosphate levels. Vascular calcification is a major contributor to cardiovascular injury and high mortality rates in CKD patients. Therefore, most animal and human studies have focused on the vasculature when describing ectopic calcifications and on the pathologic actions of elevated phosphate on vascular smooth muscle cells in this process. The extent of calcifications within soft tissues beyond the vasculature is not well described, and the involvement of cell types other than vascular smooth muscle cells is not clear. Here we provide a summary of CKD-associated extravascular calcifications in various tissues, which includes the lung, the gastrointestinal system, the liver, the skin, and the brain. Since phosphate elevations and widespread ectopic calcifications do not only occur in the context of CKD, but also in rare genetic disorders that affect the regulators of phosphate metabolism, the cellular transporters of phosphate and the factors protecting from mineral depositions outside of bone, we also discuss these pathologic scenarios. We describe different types of ectopic calcification to flesh out common aspects as well as differences in the potential mechanisms and target cell types. We postulate that phosphate elevations might act in various ways and on various tissues, which together causes a wide spectrum of phosphate-induced pathologies in CKD.

## Introduction

Bone calcification is the process of depositing calcium and phosphate minerals into bone tissue, which is a normal physiological process that drives bone formation and remodeling [1]. However, under certain pathologic conditions, calcium-phosphate crystals can also be deposited outside of the skeletal system in various soft tissues, a process that is called ectopic calcification, extraosseous or extraskeletal calcification, or calcinosis [2–4]. Ectopic calcifications include calcifications of the vasculature, which are common in patients with chronic kidney disease (CKD), where they contribute to cardiovascular events and high mortality rates [5, 6]. Vascular calcification is a cell-mediated process that is based on the activation of osteogenic gene programs in vascular smooth muscle cells in the vascular wall and their transdifferentiation into osteoblast-like cells [7]. Cellular changes include alterations in the extracellular matrix, which then promotes the precipitation of calcium-phosphate crystals to form hydroxyapatite [7]. Elevated serum phosphate (also called hyperphosphatemia) is a core parameter of CKD that has been identified as a major driver of vascular calcification in CKD, as discussed by many review articles [5, 7–9]. Elevated phosphate can directly targeting vascular smooth muscle cells and induce osteogenic differentiation [9]. Furthermore, high phosphate levels can induce a pro-inflammatory environment, which synergistically with the transdifferentiated vascular smooth muscle cells drives the calcification process [10, 11].

Interestingly, in CKD the mineralization of the vascular tree is accompanied by bone demineralization, also called the “calcification paradox”, which seems to be also driven by high phosphate levels [12]. Furthermore, pathologic actions of elevated phosphate on the heart [13, 14], skeletal muscle [15] and kidney [16, 17] have been discussed elsewhere. Briefly, high phosphate levels might directly damage the heart in multiple ways, not just by causing calcification of the coronary arteries and valves [18, 19], but also by inducing myocardial calcification and pathologic cardiac remodeling [20, 21]. Skeletal muscle tissue does not seem to undergo calcification in CKD, and phosphate might rather contribute to CKD-associated atrophy of myofibers and sarcopenia [22]. For the kidney, hyperphosphatemia might not only be a consequence of reduced renal function, but also contribute to the progression of kidney injury, which includes the formation of calcium-phosphate crystals in the tubular lumen [16]. These crystals can damage tubular epithelial cells and might also induce specific signaling events that further drive kidney damage, including local inflammation [23, 24]. Here, we focus on CKD-associated calcification in tissues other than the vasculature, heart, skeletal muscle and kidney. Although ectopic calcifications are a pathological phenomenon that is associated with hyperphosphatemia and that often occurs in various soft tissues during late stages of CKD [2], most animal and human studies focus on the vasculature when describing soft tissue calcifications. The extent of calcifications within soft tissues, beyond blood vessels, has been only poorly described and the involvement of other cell types than vascular smooth muscle cells is not well understood. In general, it has been thought that only cells of mesenchymal origin, such has osteoblasts or vascular smooth muscle cells, can transdifferentiate into an osteogenic phenotype and calcify [25]. Here, we discuss the potential contribution of different cell types to extravascular calcifications in soft tissues.

## Potential mechanisms and therapeutic targets of ectopic calcification

Inorganic phosphate is an essential mineral that is required by every cell type in the body and involved in various cellular functions and structures [26]. Dietary phosphate is taken up in the small intestine through the sodium-dependent phosphate transporter, NaPi-2b, and through paracellular transport [27]. Phosphate is distributed via the circulation and taken up by cells via two sodium-dependent phosphate transporters, called PiT-1 and PiT-2, to support cellular house-keeping functions. Most of the body’s phosphate is found in the extracellular matrix of bones in form of hydroxyapatite. Excess phosphate is released from the body by renal excretion. The kidneys can reuptake filtered phosphate via NaPi-2a and NaPi-2c and thereby preserve phosphate [27]. Phosphate homeostasis is mainly regulated by three endocrine factors, i.e. fibroblast growth factor 23 (FGF23), parathyroid hormone (PTH) and vitamin D, which provide communication between the intestine, bone, and kidney [5, 28]. PTH and FGF23 target proximal tubular epithelial cells via PTH receptors (PTHR) and FGF receptor (FGFR)/klotho co-receptor complexes, respectively, and reduce surface expression of NaPi-2a and NaPi-2c, thereby lowering renal phosphate reabsorption and serum phosphate levels. In contrast, vitamin D increases phosphate uptake in the gut by upregulating NaPi-2b in enterocytes resulting in increased serum phosphate concentrations. In CKD, decreased kidney function and reduced responsiveness to the endocrine regulators result in hyperphosphatemia, that is accompanied by compensatory increases in serum levels of FGF23 and PTH and decreases in vitamin D [5, 29]. Reductions in kidney function also cause the development of hyperphosphatemia during the normal aging process [30]. Furthermore, high dietary phosphate intake can significantly increase serum phosphate levels for several hours [31, 32], indicating that hyperphosphatemia can also occur in healthy individuals.

Controlled phosphate homeostasis is necessary to ensure that all cells in the body are provided with sufficient amounts of phosphate, while significant elevations of serum phosphate concentrations are avoided, since high phosphate has various pathologic effects [33, 34]. To date, vascular calcification is the best understood pathologic event associated with hyperphosphatemia [7, 8, 35]. However, while the cellular changes in vascular smooth muscle cells have been well-described [7], it is still unclear in what form and through which mechanisms and targets elevated phosphate can damage cells and tissues [36]. Rises in serum phosphate levels can result in the formation of insoluble calcium-phosphate crystals, which can grow and eventually precipitate in tissues as hydroxyapatite [37]. The generation of solid particles might underlie some of the pathologic actions of hyperphosphatemia in CKD patients, as suggested by experimental studies [38–40]. The formation of calcium-phosphate crystals is a chemical reaction that can occur in the absence of cells and that is regulated by the concentration of the substrates (i.e. phosphate and calcium), temperature and pH [41, 42]. For example, an acidic pH blocks crystal formation, and animal studies have shown that urine acidification by ammonium treatment reduces the amount of calcium-phosphate crystals in the renal tubular fluid [23]. This finding might have importsnt clinical impact, as metabolic acidosis is common in CKD patients and tackled by bicarbonate treatment [43]. It is possible that correcting metabolic acidosis might have disadvantages caused by urine alkalization that facilitates the formation of calcium-phosphate crystals in the tubular lumen.

**Fig. 1:**
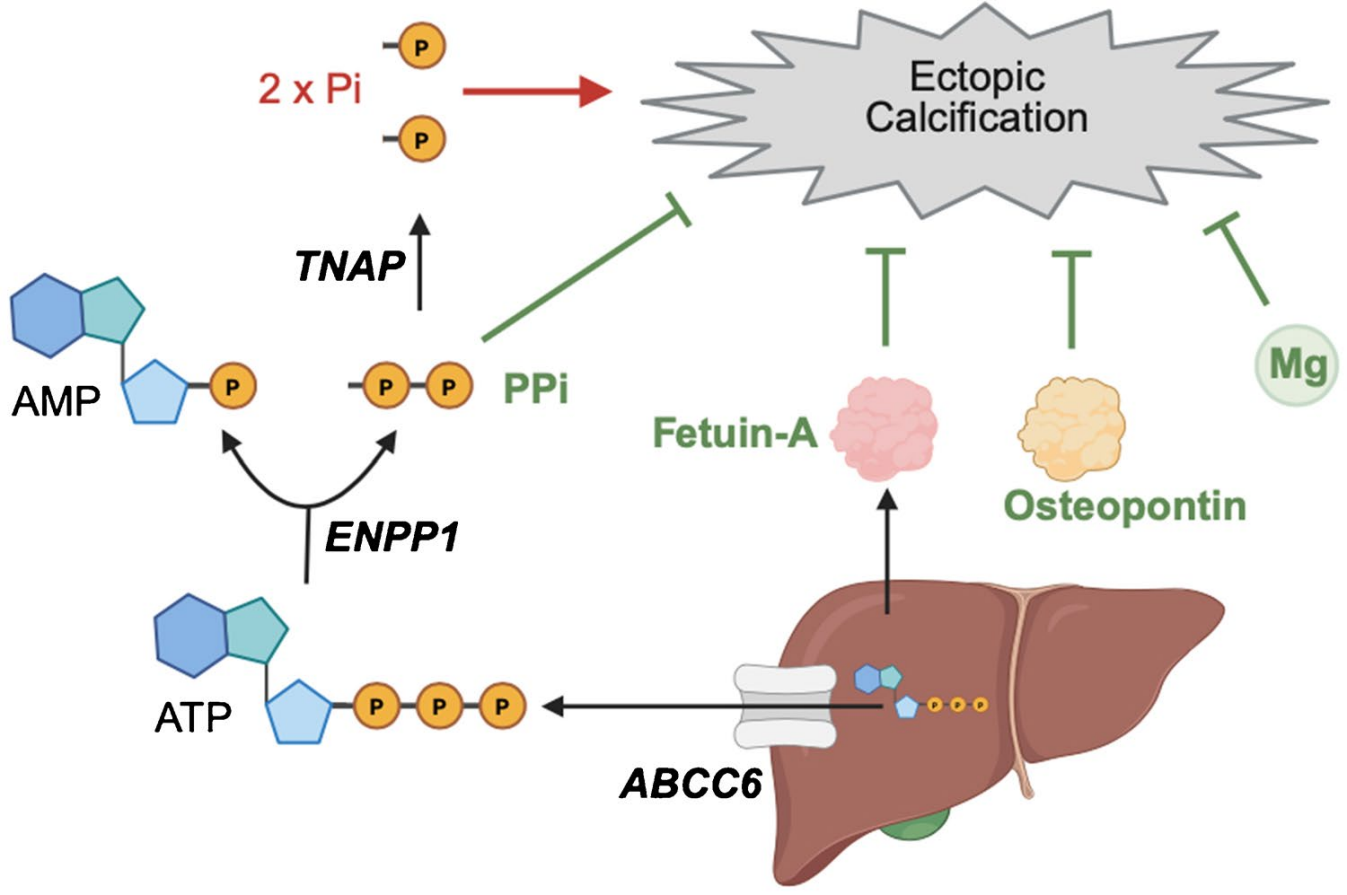
The mechanisms protecting from ectopic calcification. Inorganic phosphate (Pi) is a major inducer of ectopic calcification, whereas pyrophosphate (PPi), magnesium (Mg), fetuin-A and osteopontin are potent inhibitors of ectopic calcification. PPi and Mg prevent the initial chemical reaction of forming calcium-phosphate particles, whereas fetuin-A and osteopontin bind calcium-phosphate particles to prevent their maturation into crystals. Osteopontin is produced by different cell types and acts locally, while fetuin-A is mainly produced by the liver and acts systemically. PPi is produced in a multistep process, involving ATP Binding Cassette Subfamily C Member 6 (ABCC6) in the liver, which mediates the export of ATP and thereby increases systemic ATP levels. Ectonucleotide pyrophosphatase/phosphodiesterase 1 (ENPP1) that is expressed on the surface of various cell types, hydrolyzes ATP to AMP and PPi. Through their combined action of elevating systemic PPi levels, ABCC6 and ENPP1 contribute to the protection from ectopic calcification. Tissue-nonspecific alkaline phosphatase (TNAP) is also expressed on the surface of many cell types and hydrolyzes PPi to Pi, thereby reducing an inhibitor and generating a driver of calcification

Insoluble crystals can harm cells in various ways [44]. However, in healthy conditions, elevations in serum phosphate levels do not simply lead to the uncontrolled formation of calcium-phosphate crystals outside of the bone, such as in the blood or in soft tissues, which is based on the presence of a multi-layered safety net (Fig. 1) [1]. Magnesium and pyrophosphate (PPi) act as calcification inhibitors by blocking the chemical reaction of calcium-phosphate crystal formation [45, 46]. Similar to phosphate, magnesium is an essential mineral taken up with the diet, and systemic magnesium levels are regulated by intestinal absorption, bone exchange and renal excretion [47]. PPi is a metabolite that is generated by many cells [1, 45]. Extracellular ATP that is released from the liver via ATP Binding Cassette Subfamily C Member 6 (ABCC6) serves as the major source of systemic PPi [48, 49]. Ectonucleotide pyrophosphatase/phosphodiesterase 1 (ENPP1) is transmembrane protein expressed by various cell types that rapidly hydrolyzes extracellular ATP into AMP and PPi, thereby acting as the primary producer of extracellular PPi in the body [50, 51]. Tissue-nonspecific alkaline phosphatase (TNAP) is a ubiquitously expressed enzyme that is anchored on the outer side of the cell membrane and that hydrolyzes PPi, thereby reducing local PPi and elevating phosphate levels [52].

CKD is a state of low systemic levels of PPi and magnesium [45, 53]. It has been shown that injections of PPi [54, 55] or elevating magnesium levels by the administration of a high-magnesium diet [46, 56, 57] protects animal models of CKD from developing vascular calcification. However, the therapeutic value of these findings in CKD is not clear. While bisphosphonates, which are non-hydrolyzable analogs of PPi, are widely used for the treatment of osteoporosis, they adversely affect skeletal biology and are cleared by the kidney, which limits their use in CKD [58]. Various clinical studies have aimed to determine the beneficial effects of magnesium elevations in patients with CKD [59]. Unfortunately, two major clinical trials in pre-dialysis CKD patients that have examined the effects of oral magnesium supplementation produced contradictory findings on the progression of coronary artery calcification [60, 61]. However, small clinical studies in patients with end-stage renal disease (ESRD) receiving hemodialysis with increases in the dialysate magnesium concentration reported encouraging results, including reductions in the propensity to form calcium-phosphate crystals in the circulation and in all-cause mortality [62–64]. A pragmatic trial in about 25,000 dialysis patients, that is currently underway in Canada to examine the effects of dialysate magnesium on clinical outcomes, should provide further important insights into the therapeutic value of elevating magnesium levels in CKD (clinicaltrials.gov; NCT04079582).

While magnesium and PPi prevent the chemical reaction of crystal formation, the protein fetuin-A acts as a sponge that binds calcium and phosphate to form calciprotein particles (CPPs) thereby preventing the growth and maturation of crystals [65, 66]. In early stages, CPPs are amorphous and dispersed in solution and can be filtered and removed by the kidney via megalin-mediated endocytosis in the proximal tubule [39, 67]. In later stages, solid CPPs are cleared rapidly from the circulation by sinusoidal endothelial cells and Kupffer cells in the liver and by macrophages in the spleen [41, 67–70]. Fetuin-A is mainly produced by the liver, and as a circulating calcium-phosphate buffer fetuin-A acts as a systemic inhibitor of soft tissue calcification (Fig. 1). CKD is a state of low systemic levels of fetuin-A [71–73], which could contribute to ectopic calcifications. Therapeutic approaches to elevate fetuin-A have been tested for other diseases, and they might also have beneficial effects in CKD, which needs to be determined [71]. However, since fetuin-A has biological activities beyond the inhibition of calcification, such as the regulation of the immune system, the inflammatory response, and glucose metabolism, fetuin-A might have various effects which require a fine balance of fetuin-a levels [71]. Osteopontin, which is produced by various tissues, is a multifunctional protein that has similar calcium-phosphate-buffering functions like fetuin-A [74, 75], and acts mainly locally to inhibit mineral deposition and to regress calcification [76, 77]. Different from fetuin-A, the levels of osteopontin rise in CKD [73, 78], potentially as a direct protective response to elevations in levels of phosphate and CPPs [23, 24, 79, 80]. Various other endogenous inhibitors of calcification have been described, including GLA proteins, sclerostin, and osteoprotegerin, which also seem to be dysregulated in CKD [1, 81, 82].

**Fig. 2:**
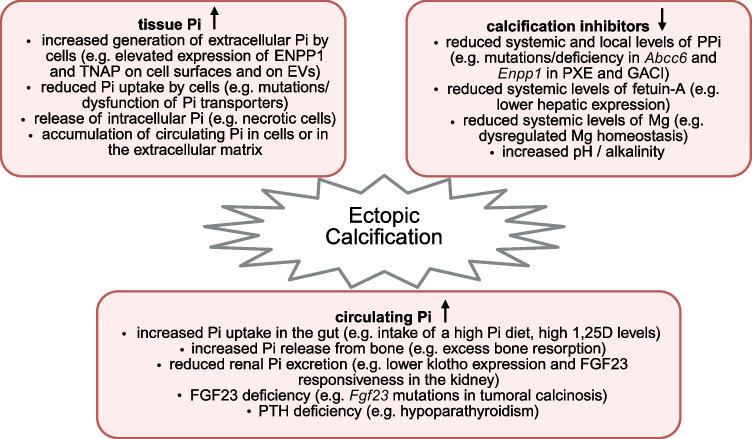
The drivers of ectopic calcification. Elevations in the levels of inorganic phosphate (Pi) in the circulation and in tissue can both cause ectopic calcifications. However, increases in serum and tissue Pi levels seem to have different causes. Since local mechanisms control Pi concentrations in the surroundings of cells, it appears that increases in tissue Pi levels do not necessarily have to be accompanied by elevations in circulating Pi levels. Mutations or other pathologic events leading to a loss, reduction or dysfunction of the inhibitors of calcium-Pi crystal formation also result in ectopic calcification that can occur in the presence of normal Pi levels. CKD is accompanied by events leading to elevations in circulating Pi and reductions in calcification inhibitors. Whether CKD also results in the increase in tissue Pi content prior to the appearance of calcifications needs to be established. It is plausible to assume that ectopic calcification is most severe and detrimental when all three components are altered, i.e. high levels of serum and tissue Pi and low levels of inhibitors

Overall, while various events can contribute to ectopic calcifications, it is the co-appearance of these events that together induce and drive severe calcifications, as the case in CKD. These events include reductions in the inhibitors of calcification combined with elevations in circulating and local levels of phosphate (Fig. 2) [83]. Therefore, a combination of therapeutic approaches might be needed to efficiently tackle CKD-associated ectopic calcifications.

## Genetic mouse models of ectopic calcification

Not surprisingly, the loss of mechanisms protecting from calcium-phosphate crystal formation result in ectopic calcifications. For example, mice with *Abcc6* deletion have reduced serum PPi levels [84], and they spontaneously develop calcifications in various tissues [85]. In humans, dystrophic cardiac calcinosis (DCC) is a genetic disorder caused by mutations in *Abcc6* with ectopic calcifications in various tissues, including the heart [86, 87]. Pseudoxanthoma elasticum (PXE) is an autosomal recessive disease with *Abcc6* deficiency resulting in reduced serum PPi levels, elevations in calcium and phosphate levels and progressive ectopic calcifications [88–90]. Furthermore, some cases of generalized arterial calcification of infancy (GACI), which is a rare genetic disease with early-onset and life-threating ectopic calcifications, are caused by *Abcc6* mutations [90]. Mouse models with genetic ENPP1 deletion (*Enpp1*^*−/−*^) or carrying a missense mutation for *Enpp1* have reduced PPi levels and are characterized by extensive calcifications in multiple organs [91–95]. Calcifications worsen when mice are administered a combined high-phosphate and low-magnesium diet [91]. The majority of patients with GACI have *Enpp1* mutations leading to ENPP1 dysfunction [51]. Transgenic mice overexpressing TNAP in endothelial cells (*TNAP-Tg*), which reduces PPi levels and increases local levels of phosphate, develop sever calcifications in the vasculature and in many tissues [96]. To date, mutations leading to TNAP overexpression or overactivation have not been reported in humans. Combined, these studies show that reductions in PPi levels cause severe calcifications in various tissues.

Soft tissue calcifications have been also detected in mice with deletion of fetuin-A (*Ahsg*^*−/−*^), but only when administered a high-phosphate/high-vitamin D diet or when done in the calcification-prone DBA/2 genetic background [97, 98]. Vitamin D increases intestinal absorption of phosphate and calcium thereby elevating serum levels of phosphate and calcium. Furthermore, it appears that a hypomorphic *Abcc6* mutation in the inbred DBA/2 strain contributes to its high susceptibility for soft tissue calcifications [99]. A longitudinal analysis of this mouse model suggests that soft tissue calcifications start with intravascular mineral depositions which cause microvasculopathy and impact organ function, rather than osteogenic cell differentiation within the tissue [97]. Soft tissue calcifications in *Ahsg*^*−/−*^ mice seem to be based on the loss of fetuin-A’s inhibitory actions on CPP formation. Furthermore, *Ahsg*^*−/−*^ mice in the DBA/2 background not only lack fetuin-A but also have reduced levels of magnesium and PPi, therefore having low levels of three major inhibitors of extracellular mineralization [99]. Administration of fetuin-A or PPi or a magnesium-rich or low-phosphate diet reduces soft tissue calcifications in *Ahsg*^*−/−*^ mice [99]. To date, only one case of a *Ahsg* mutation leading to fetuin-A deficiency has been reported, where the patient developed bone hyperplasia during infancy, but soft tissue calcifications were not described [100]. Vascular and renal calcifications have been also reported in mice with global deletion of osteopontin (*Spp1*^*−/−*^) following the administration of a high-phosphate diet or the induction of CKD [24, 101].

It is also not surprising that impairments in the phosphaturic FGF23-klotho system lead to hyperphosphatemia, as shown in genetic mouse models that lack FGF23 (*Fgf23*^*−/−*^) or klotho (*kl/kl*), which both develop severe ectopic calcifications in the absence of kidney injury [102–105]. Interestingly, NaPi-2a deletion (*NaPi-2a*^*−/−*^) in *kl/kl* mice, which lowers serum phosphate levels, protects from ectopic calcifications, and calcifications appear again when *kl/kl;NaPi-2a*^*−/−*^ mice are administered a high-phosphate diet [105, 106]. These findings suggest that hyperphosphatemia drives ectopic calcifications in *kl/kl* mice. Of note, different from CKD, *Fgf23*^*−/−*^ and *kl/kl* mice also have elevated serum levels of vitamin D and calcium [103, 107], which further increase systemic phosphate levels and the formation of calcium-phosphate crystals [104, 108, 109]. Moreover, an alkaline environment seems to promote ectopic calcifications in *kl/kl* mice, since the induction of acidosis has protective effects [110–112]. Surprisingly, *kl/kl* mice have elevated serum levels of fetuin-A and osteopontin [112], and their anti-calcification activities seem to fail in this mouse model. In humans, genetic deficiencies of *klotho* and of *Fgf23* as well as the regulators of FGF23 production cause familial tumoral calcinosis [113–116], which is a rare genetic disease with hyperphosphatemia and ectopic calcifications [117]. FGF23 signals in the kidney via FGFRs, and hyperphosphatemic tumoral calcinosis has been described in cancer patient receiving an inhibitor against FGFRs as chemotherapy [118]. Rats receiving a FGFR inhibitor develop widespread ectopic calcifications [119], most likely based on the loss of FGF23’s phosphaturic actions on the kidney.

Overall, studying genetic diseases and animal models with a loss of calcification inhibitors or phosphate regulators should provide important insights into the causative role of elevated phosphate in ectopic calcifications. While CKD is a complicated multifactorial disease, a better understanding of these genetic models might provide important insights into the pathomechanisms underlying CKD-associated soft tissue calcifications.

## Types of ectopic calcifications

**Fig. 3:**
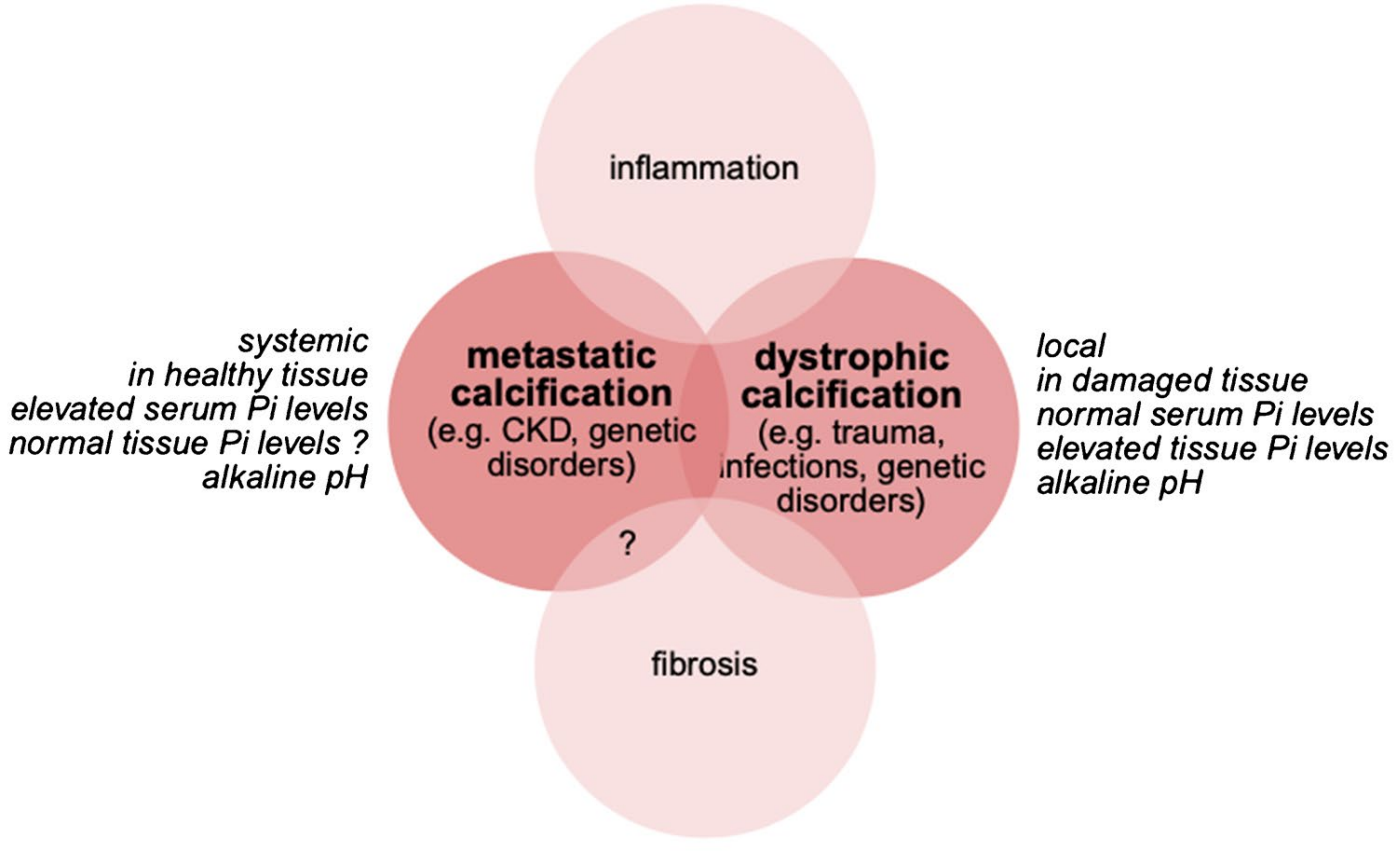
The different types of ectopic calcification. Metastatic and dystrophic calcification have been described as separate pathologic events, since they have different causes and mechanisms, and they are associated with different diseases. However, we propose that CKD that is traditionally seen as a scenario of metastatic calcification with elevations in serum phosphate (Pi) levels, is also accompanied by elevations in tissue Pi levels, which has been traditionally linked to dystrophic calcification. Therefore, CKD might resemble both, metastatic and dystrophic calcification, which might be driven by different pathomechanisms. Metastatic and dystrophic calcification occur in the context of systemic and local inflammation. While damaged tissues, including fibrotic areas, acts as the sites of dystrophic calcification, it is unclear if metastatic calcification occurs in the context of fibrosis. The crosstalk between ectopic calcification, inflammation and fibrosis and the precise order of events needs further investigation. Furthermore, the molecular and cellular targets of elevated Pi within this pathologic network need to be identified

Ectopic calcifications can be categorized into events that occur systemically or locally (Fig. 3). Metastatic calcifications are precipitations of calcium-phosphate crystals that are not isolated but that can occur in normal tissue anywhere throughout the body [120–123]. Metastatic calcifications are more prevalent in tissues with increased alkalinity, including the gastric mucosa, kidney, lung, systemic arteries and pulmonary veins [120, 122, 124]. Furthermore, various epithelial secretions like saliva and alveolar surfactant are rich in phosphate, conferring some inherent risk of calcium-phosphate precipitations [67]. Metastatic calcification usually results from an underlying disorder of systemic calcium-phosphate homeostasis and is caused by elevations in serum levels of calcium and/or phosphate. In contrast, dystrophic calcification occurs at sites of pre-existing tissue damage and seems to be independent of systemic alterations in calcium-phosphate homeostasis [4]. This local calcification process is probably a consequence of cell death, with the release of intracellular calcium, phosphate and alkaline phosphatase, and an increase of the local pH, which all together result in the precipitation of calcium-phosphate crystals. Traditionally, metastatic and dystrophic calcifications are seen as separate events driven by different causes and mechanisms [1, 2]. However, more recent studies suggest that dystrophic calcification is not simply based on local changes in the chemical parameters of calcification caused by dying cells, but also by changes in the enzymatic activity of living cells, including increased cell surface expression of ENPP1 and TNAP, that elevate local phosphate concentrations in the calcifying areas [21]. Such local and cell-based mechanisms of calcification have been also described in CKD-associated vascular calcification, where besides the systemic phosphate elevations also local phosphate increases seem to contribute to the calcification event [7]. It is possible that mechanisms driving dystrophic calcification come into play in scenarios of metastatic calcification (Fig. 3), such as CKD, and that not only elevations of circulating but also of local levels of phosphate contribute to ectopic calcification in CKD (Fig. 2).

Ectopic calcification is not an isolated event but occurs in the context of other histological changes (Fig. 3). For example, elevated phosphate and CPPs can induce an inflammatory response systemically and locally [10], and the appearance of insoluble crystals and soft tissue calcifications seems to be commonly accompanied by inflammation [44]. In some cases, inflammatory cells, such has monocytes, themselves can undergo osteogenic differentiation and calcify [125]. However, the precise order of events and the molecular nature of the crosstalk between calcification and inflammation still needs clarification. Dystrophic calcification of soft tissues usually occurs in areas of injury associated with fibrosis [126], and an experimental study has shown that cardiac fibroblasts can adopt osteoblast cell-like fates and directly contribute to calcifications in the heart muscle [21]. Whether fibroblasts also contribute to metastatic calcifications in the absence of injury and in various tissues is currently not known [127]. Furthermore, it is unclear whether elevated phosphate can cause fibrosis. Inflammation and fibrosis are initially compensatory and protective mechanisms that contribute to tissue maintenance and regeneration, and that only after sustained activation turn into pathologic events that can worsen tissue damage [128]. It is possible that soft tissue calcification has similar adaptive features [11]. However, potential beneficial effects of ectopic calcification remain to be explored.

## Phosphate and lung

Lung disease is increasingly recognized to contribute to the morbidity and mortality in patients with kidney disease [129]. Clinical studies have shown that not only patients with acute kidney injury (AKI) [130–133], but also with CKD [134–144] have a significantly higher susceptibility to develop lung injury. Compared to individuals without kidney disease patients with CKD are more likely to die from lung disease [144]. In CKD, various pathologic consequences are shared between the lung and kidney, including tissue inflammation, fibrosis and edema [129]. CKD and lung disease also share common risk factors, such as smoking [145, 146] and age [147], but the mechanisms that contribute to CKD-associated lung disease are largely unknown. While lung injury has been studied in animal models of AKI [132], only a few models of CKD have been analyzed for their lung phenotype. Mice fed an adenine-rich diet for several weeks and mice with global deletion of the collagen type IV alpha 3 chain (*Col4a3*^*−/−*^), which are both models of progressive CKD, develop lung inflammation and injury, with secretion of inflammatory cytokines by various cell types, including bronchial epithelial cells, and leukocyte infiltration in the alveolar and bronchial walls [148, 149]. An analysis of lung dysfunction and its association with inflammation across different stages of CKD revealed that inflammation is a key factor in the kidney-lung interaction and might play an important role in the pathogenesis of respiratory damage in CKD [137]. Interestingly, elevated serum phosphate levels have been associated with chronic obstructive pulmonary disease (COPD) and mortality in the elderly [150]. However, whether hyperphosphatemia also correlates with lung injury in CKD patients and whether elevated phosphate levels can contribute to lung injury is currently unknown.

It is unclear whether CKD-associated ectopic calcifications occur in the lung. In fact, pulmonary calcification is rarely diagnosed in CKD patients during life; however, this could be due to the poor sensitivity of standard chest radiographs for the identification of calcifications and the lack of awareness among clinicians of pulmonary calcifications. Interestingly, a study using dual-energy digital chest radiography detected increased calcium content in the lung of dialysis patients [151]. In contrast, soft tissue calcifications can be visualized by high-resolution computed tomography (CT) [152], and a high-resolution CT study that included CKD patients reported pulmonary calcifications that were biopsy-proven [153]. Furthermore, some autopsy studies have identified pulmonary calcifications in dialysis patients [121, 151, 152, 154–158]. Combined these studies suggest that about 75% of hemodialysis patients develop pulmonary calcifications, and then mainly in the alveolar walls [159]. Pulmonary calcifications have been also described in other context than CKD, such as primary hyperparathyroidism, excessive administration of calcium or vitamin D, lung infections, liver transplantation and heart surgery [154]. It seems that hypercalcemia, a local alkaline environment and previous lung injury predispose for pulmonary calcification [154]. In general, it appears that pulmonary calcification mainly occurs in the alveoli [155, 160], which might be driven by the alveolar surfactant which per se is rich in phosphate based on the presence of phosphate-containing lipids [161].

A recent study in miniature pigs with bilateral nephrectomy identified calcifications in the lung, and mainly in the alveolar walls, as determined by 3D-CT imaging [162]. Removal of CPPs from the blood using an adsorption column during the hemodialysis process, improved survival and alleviated pulmonary and vascular calcification as well as chronic inflammation in nephrectomized pigs [162], suggesting that CPPs cause pulmonary calcification. Furthermore, a recent study in the adenine mouse model of CKD has shown that acute phosphate loading results in tissue-specific phosphate depositions, with highest accumulations in the vasculature and significant phosphate depositions in some other tissues, including the lung [163]. This study suggests that the non‐renal clearance of excess phosphate might cause the accumulation of phosphate in the lung, among other tissues, and drives tissue calcification [34]. Interestingly, a recent study in patients with systemic sclerosis, a complex auto-immune disease with calcium deposits in the lung and with mortality mainly caused by pulmonary events [164], has shown that reduced lung function is associated with increased serum CPP levels [165]. However, whether CPPs cause lung calcification in systemic sclerosis needs to be determined.

Studies in non-CKD animal models with hyperphosphatemia further suggest that phosphate can cause lung damage, including calcifications. For example, *Fgf23*^*−/−*^ mice develop lung emphysema and pulmonary calcifications [104, 166–169]. When *NaPi-2a* was deleted in *Fgf23*^*−/−*^ mice to lower renal phosphate reuptake, serum phosphate levels were reduced and lung emphysema were ameliorated [167], suggesting that high phosphate contributes to lung injury in mice lacking FGF23. Similarly, *kl/kl* mice develop lung emphysema with an enlargement of air spaces associated with alveolar destruction [102, 105, 106, 110, 111, 168, 170–184], calcification of lung [105, 110–112, 171, 176, 177] and trachea [110–112, 176, 177], as well as reduced lung function [179]. *NaPi-2a* deletion in *kl/kl* mice, which lowers serum phosphate levels, protects from lung emphysema and calcification, and these pathologies appear again when double-knockout mice were put on a high-phosphate diet [105, 106], suggesting that hyperphosphatemia drives lung damage in *kl/kl* mice. In the 1960s, the administration of inorganic phosphate, either through intravenous infusion or oral supplementation, was used as a therapeutic strategy to treat hypercalcemia, which provided evidence for the pathologic effects of elevated phosphate in humans. Some of these patients developed pulmonary edema, and autopsies revealed ectopic calcifications which were mainly located in the lung, heart and kidney [185]. Furthermore, phosphate solutions have been widely used as an enema, especially for the treatment of constipation and preoperative bowel preparation in children. Phosphate enemas have been reported to cause breathing difficulties and respiratory disorders [186, 187].

Although the precise target cells that contribute to lung calcifications remain unclear, cell culture studies have shown that phosphate elevations can have direct pathologic actions on various cell types. For example, in human bronchial epithelial cells, phosphate induces the secretion of inflammatory cytokines, which is mediated by Ras/mitogen-activated protein kinase (MAPK) signaling [149], as well as cell proliferation [188]. Furthermore, treatment of human alveolar epithelial cells with high phosphate induces oxidative stress and apoptosis [174]. Whether alveolar epithelial cells can also calcify and contribute to lung calcifications in CKD, as suggests by the identification of alveolar calcification in histological studies in patients and mouse models, needs to be determined. Interestingly, a tumor cell line of alveolar epithelial cells shows osteoblast-like features, suggesting that in lung cancer alveolar epithelial cells might cause pulmonary calcification [189]. In general, pulmonary calcifications have been described in patients with certain types of cancer, including myelomas and leukemias [190–194].

As mentioned earlier, mouse models with reductions in calcification inhibitors develop widespread soft tissue calcifications, which can also include the lung. For example, mice with *Abcc6* deficiency and low serum PPi levels develop not just DCC but also alveolar calcifications [85]. Mouse models with *Enpp1* deficiency also have reduced PPi levels and develop pulmonary calcifications [91, 95], which worsens when mice are administered a combined high-phosphate and low-magnesium diet [91]. However, only a few studies have described pulmonary calcifications in patients with PXE [195], and lung calcifications, other than calcification of the pulmonary arteries [196], have not been reported in GACI. *TNAP-Tg* mice with reduced PPi levels and increased local levels of phosphate, develop sever calcifications in the lung, which is detectable by histological analysis [96]. Pulmonary calcifications have been also observed by histological stainings and CT scans in *Ahsg*^*−/−*^ mice that received a high phosphate/high-vitamin D diet or a high-phosphate diet combined with the induction of CKD [97, 98, 197]. Administration of fetuin-A or PPi or a magnesium-rich or low-phosphate diet reduces pulmonary calcification in *Ahsg*^*−/−*^ mice [99].

The formation of small calcium-phosphate deposits, also called microliths, in the alveoli have been described in pulmonary alveolar microlithiasis (PAM), which is a rare autosomal recessive disease [150, 198]. Usually, the disease progresses slowly and often leads to respiratory failure. Currently, lung transplantation is the only effective treatment [199]. PAM is caused by mutations in *NaPi-2b*, which is expressed in several cell types and organs, including type II alveolar cells in the lung [200–205]. Since the identification of *NaPi-2b* as a disease causative gene in 2006 [206, 207], 30 allelic variants have been reported in PAM patients [208]. The deletion of *NaPi-2b* in the lung epithelium in mice (*NaPi-2b*^*−/−*^) results in a PAM-like phenotype, including alveolar microlith accumulation as well as calcification, inflammation and fibrosis in the lung tissue [209]. Similarly, the inducible global deletion of *NaPi-2b* results in lung calcifications [200]. Interestingly, while lung calcification in *NaPi-2b*^*−/−*^ mice occurs in the absence of elevations in serum phosphate levels [200], administration of a low phosphate diet to these mice reduces alveolar microlith accumulation and lung injury [209]. The precise role of NaPi-2b in alveoli is not clear, but primary cultures of type II alveolar epithelial cells have been shown to take up phosphate via sodium-dependent transport [210, 211]. NaPi-2b may play an important role in the regulation of phosphate levels in the fluid lining the alveolar space [203, 209]. Phosphate-containing lipids are a component of surfactant [161], and surfactant synthesis might be regulated by NaPi-2b-mediated alveolar phosphate uptake. The dysfunction of mutated NaPi-2b might lead to decreased cellular uptake of phosphate and increased phosphate levels in the alveolar space followed by the precipitation with extracellular calcium and crystal formation [206]. This hypothesis is supported by in vitro studies. Phosphate uptake experiments conducted in *Xenopus laevis* oocytes have shown that some of the overexpressed NaPi-2b PAM variants were non-functional [206, 212]. Furthermore, a NaPi-2b mutant variant overexpressed in a human alveolar epithelial cell line revealed evidence of dysfunctional phosphate transport [213]. A more recent study suggests that the PAM pathomechanism might also include the osteoclast-like differentiation and activation of alveolar monocytes [125]. Interestingly, the transfer of microliths isolated by bronchoalveolar lavage from *NaPi-2b*^*−/−*^ into the lungs of healthy wildtype mice results in PAM-like injury [200], suggesting that microliths cause the observed lung injury.

Overall, it appears that hyperphosphatemia can drive pulmonary calcification, but future studies need to identify the precise pathologic context, the cell types involved and the underlying mechanisms. Additional clinical studies are needed to determine if pulmonary calcification is indeed a significant CKD-associated pathology that might contribute to overall morbidity and mortality. In general, the impact of alveolar calcification on lung function needs to be established. Pulmonary calcification often seems to be asymptomatic and not affect lung function, but it can potentially progress to respiratory failure [159]. It is likely that the alkaline environment in the lung promotes the precipitation of calcium-phosphate crystals, suggesting that the calcification process in the lung might not only be a regulated cell-mediated process, but also a chemical reaction.

## Phosphate and liver

Kidney and liver disease can occur together, also called hepatorenal syndrome [214]. However, while the contributions of advanced liver disease to the induction and progression of kidney damage are well described, it is unclear if and how primary CKD can cause liver disease. It appears that CKD does not have major impact on liver structure, and animal models of CKD do not show significant liver pathologies. To date, hepatic calcifications have not been described in CKD. However, calcifications in the liver can occur in the context of infections and inflammation or following liver transplantation [215, 216]. Furthermore, some of the described genetic mouse models with widespread ectopic calcifications also show calcifications in the liver. This is the case for *Enpp1* deficient and *TNAP-Tg* mice which were described earlier [91, 96]. However, hepatic calcifications have not been reported in patients with PXE or GACI.

Although elevated phosphate levels might not necessarily damage the liver structure, they could modify liver function, which could contribute to CKD-associated pathologies. An in vitro study in hepatocytes indicates that phosphate elevations induce the production of interleukin (IL)−6 and IL-1β which increases hepcidin expression [217]. It is well described that CKD is associated with systemic inflammation and anemia [218, 219], and hyperphosphatemia in mice, either caused by the administration of an adenine-rich diet to induce CKD or by dietary phosphate excess, increases inflammation and exacerbates anemia [217]. In this study, liver levels of phosphate and of hepcidin expression showed a positive correlation, and the onset of increases in hepatic phosphate content preceded significant elevations in serum phosphate levels. Supplying a low phosphate diet to *Col4a3*^*−/−*^ mice resulted in beneficial outcomes that reduced functional iron deficiency [217]. In a different study the administration of a high phosphate diet to rats increased hepcidin expression and iron content in the liver [220]. These studies suggest that by targeting the liver, hyperphosphatemia might contribute to systemic inflammation and induce anemia. It has been shown in patients with CKD that hyperphosphatemia is associated with inflammation, anemia and mortality [221, 222]. The association between hyperphosphatemia and anemia has been also reported in adults without CKD [223].

Feeding studies in rodents have shown that a high-phosphate diet negatively regulates lipid synthesis in the liver [224, 225]. However, mice lacking the phosphate transporter PiT-1 specifically in hepatocytes show decreased lipogenesis, which also results in increased glucose tolerance and insulin sensitivity and protection against high-fat diet-induced obesity and diabetes [226]. While the precise actions of phosphate and the underlying mechanisms and effects need further investigation, these animal studies suggest that phosphate might act as a metabolic regulator in the liver. Interestingly, during embryonic development global *PiT-1*^*−/−*^ mice show reduced liver growth and hematopoiesis resulting in anemia [227, 228]. Liver cells from these mice are defective in proliferation, resulting in impaired regeneration after partial hepatectomy [227]. While blocking phosphate access to the liver seems to inhibit hepatic growth, it has been shown that phosphate elevations have the opposite effect. A high phosphate diet in developing mice and rats increases cell cycle progression and angiogenesis in the liver and liver mass [229, 230]. Similar effects of high phosphate diets have been observed for the developmental growth of the lung [231] and brain [232]. Interestingly, phosphate-dependent organ growth does not seem to be mediated by changes in serum phosphate levels [233].

It is known that following hepatectomy, serum phosphate levels drop, which is due to the increased metabolic demand of the regenerating liver, but also includes increased renal phosphate excretion [234, 235], suggesting an endocrine crosstalk between liver and kidney that regulates phosphate metabolism. Phosphaturia and hypophosphatemia have been also reported in patients with acute liver failure [236, 237]. Of note, a recent experimental study suggests the existence of a nerve-mediated communication pathway between liver and kidney that acts independently of the known endocrine regulators of renal phosphate excretion, i.e. FGF23 and PTH [238]. In healthy rats the administration of radiolabeled phosphate to the duodenum resulted in phosphate uptake by the liver within minutes. In what form and where phosphate is stored in the liver, and if it can be released back into the circulation, is unclear. Through the nervous system the liver then communicates with the kidney to reduce NaPi-2a levels and thereby increases renal phosphate excretion. This study suggests that the liver acts as a sensor for elevations in serum phosphate levels in the acute postprandial phase that quickly communicates with the kidney to lower circulating phosphate concentrations [238]. If this mechanism also acts to counterbalance prolonged hyperphosphatemia, and whether it is dysregulated in CKD needs to be determined.

Overall, while hyperphosphatemia does not seem to induce structural changes and calcifications in liver tissue in CKD, it might impact the metabolic functions of the liver as well as liver growth and regeneration. Furthermore, the liver seems to communicate with the kidney to regulate renal phosphate excretion and thereby systemic phosphate levels. The physiologic significance of these findings, and whether they contribute to CKD-associated metabolic changes, needs to be determined.

## Phosphate and the gastrointestinal system

CKD patients have abnormal functions in different segments of their digestive tract, including local inflammations and calcifications [239]. Stomach biopsies from a kidney transplant patient after long-term hemodialysis, who had persistent hyperphosphatemia and hypercalcemia, detected calcium-phosphate depositions in the stomach [240]. Furthermore, an autopsy study of dialysis patients detected severe calcifications in the stomach, including mucosa, muscularis mucosa, submucosa and vessels [121]. Usually, soft tissue calcifications are found in adult CKD patients and rarely in children. However, a study in pediatric ESRD detected soft tissue calcifications in the stomach, with the gastric mucosa as one of the frequent sites of mineral deposition, among other organs [241]. Interestingly, in this study patients treated with vitamin D showed more severe calcifications. An experimental study with healthy rats treated with high-dose vitamin D resulted in hyperphosphatemia, hypercalcemia and renal injury, which was accompanied by calcifications in the mucosa and the muscularis mucosa of stomach and intestine [242]. Another experimental study in mice showed that vitamin D-treatment caused calcification in the muscularis externa, muscularis mucosa, and mucosa of the stomach, accompanied by elevated levels of phosphate, calcium and markers of kidney damage [243]. A different study in partially nephrectomized rats, a surgical model of CKD, showed elevated phosphate and calcium content and calcifications in the stomach, when animals were on a low-magnesium diet [57]. In contrast, elevating magnesium content in the diet reduced stomach calcification in these rats. As mentioned earlier, magnesium inhibits the formation of CPPs and vascular calcification [46], which appears to be also the case for calcifications in the gastrointestinal system. Furthermore, administration of a high-phosphate diet to nephrectomized rats increased calcium-phosphate content in the stomach, which was reduced when dietary phosphate uptake was inhibited pharmacologically [244].

The potential contribution of high phosphate to calcifications in the gastrointestinal system is also supported by studies in models of hyperphosphatemia without CKD. In *kl/kl* mice, calcifications have been reported in the gastric mucosa and small arteries, and potentially also in the gastric epithelial layer [102, 110–112, 170, 173], as well as in the intestinal artery walls and epithelial layer [111, 112, 176]. Calcifications in the submucosal gastrointestinal tract have been reported in patients with familial tumoral calcinosis [117]. Furthermore, *TNAP-Tg* mice develop calcifications in the intestine detectable by histological analyses [96]. Besides calcifications, high phosphate might have other pathologic actions on the gastrointestinal system. For example, intestinal inflammation and atrophy have been detected in *kl/kl* and *Fgf23*^*−/−*^ mice [104, 106, 166, 167, 182], and administration of a high-phosphate diet exacerbated intestinal inflammation in a rat model of colitis [245].

Overall, it appears that in CKD with hyperphosphatemia elevations in calcium and active vitamin D drive calcifications in the gastrointestinal system. The underlying mechanisms are not clearly defined, and targets might not only include the vasculature but also other cell types in the mucosa and submucosa. However, more studies are needed to precisely localize gastrointestinal calcifications in animal models and patients with CKD. It is important to note that a specific form of gastrointestinal calcification, called calciphylaxis, occurs in CKD. Calciphylaxis is a rare and life-threatening disorder that predominantly affects patients with ESRD [246]. While cutaneous manifestations are the most common presentation, as described below, gastrointestinal involvement can also occur. Calciphylaxis, which is also referred to as calcific uremic arteriolopathy, is based on calcium depositions in small blood vessels, resulting in vessel occlusions and subsequent ischemic tissue necrosis [246]. Interestingly, histological studies of the intestine in these patients did not only reveal extensive calcifications within arteries, but also microcalcifications within the submucosa and fat [247].

## Phosphate and skin

Calciphylaxis causes ischemic skin lesions with calcium-phosphate depositions in the dermis [2], also called cutaneous calcification or calcinosis cutis. It is found in less than 1% of ESRD patients and typically manifests as painful cutaneous lesions that progress to non-healing ulcers with superimposed infections [248]. The underlying pathogenesis is multifactorial and seems to include the dysregulated phosphate metabolism and the osteogenic differentiation of vascular smooth muscle cells [249], and therefore might be similar to the pathogenesis of medial and intimal vascular calcifications. Nephrectomized rats receiving a high-phosphate diet develop skin calcifications, which not only includes calcifications of subdermal blood vessels, but also extravascular dermal calcifications [250]. Interestingly, lowering serum phosphate levels in this model reduces skin calcifications, suggesting a causative role of phosphate in the pathologic process.

The potential contribution of high phosphate to calcifications in the skin is further supported by studies in genetic diseases and mouse models with deficiencies in calcification inhibitors. Patients with PXE and *Abcc6* deficiency develop progressive calcifications of the skin [88–90]. *Abcc6*^*−/−*^ mice and rats develop calcifications of the muzzle skin where connective tissue in the capsule of the vibrissae is affected [84, 251–254]. Injections of PPi prevent the development of skin calcifications in *Abcc6*^*−/−*^ mice [255], while dietary restriction of magnesium accelerates these calcifications [253]. Furthermore, calcifications in the dermal sheath of vibrissae have been observed in *Enpp1*^*−/−*^* mice* [94], and in mice carrying a missense mutation for *Enpp1*, which is more severe if dietary magnesium content is reduced [91, 93, 95, 256]. However, skin calcifications have not been reported in GACI. Calcifications in the skin occur in mice with deletion of *Ahsg* [97–99], which were reduced by the administration of fetuin-A or PPi or a magnesium-rich or low-phosphate diet [99]. Moreover, skin calcifications are a classic feature in patients with tumoral calcinosis [117]. Hyperphosphatemia might contribute to other skin pathologies than calcifications, as suggested by studies in *kl/kl* [102, 106, 170, 180, 182] and *Fgf23*^*−/−*^ mice [104, 166, 182], which develop skin atrophy.

The described experimental studies suggest that calcinosis cutis is accompanied by microcalcifications outside of the vasculature and that hyperphosphatemia is a contributing factor, which should be expanded on in future animal and cell culture studies. Furthermore, while skin calcifications are found in a small subset of ESRD patients, it should be determined whether they can also occur in earlier CKD stages and in larger patient populations.

## Phosphate and adipose tissue

Calcifications in adipose tissue have been reported in cases of nodular cystic fat necrosis, when necrotic adipocytes release fatty acids which then combine with calcium [257]. However, adipose calcifications have not been reported in CKD, and whether hyperphosphatemia can cause calcifications in fat tissue is unknown. Of note, CT scans of *Ahsg*^*−/−*^ mice revealed calcified lesions in brown adipose tissue (BAT), which were reduced by the administration of fetuin-A or PPi or a magnesium-rich or low-phosphate diet [97, 99]. In contrast, BAT calcifications worsened when *Ahsg*^*−/−*^ mice were put on a high-phosphate diet [99], suggesting that phosphate elevations contribute to BAT calcification in this model. Furthermore, in dialysis patients with calciphylaxis not only microvessels in the skin and in gastrointestinal system calcify, but also in subcutaneous and submucosa adipose tissue [247]. Clearly, more clinical and experimental studies are needed to determine if CKD is associated with calcifications in adipose tissue. If this is the case, it will be important to determine the type of adipose tissues that undergoes calcification as well as the metabolic consequences.

## Phosphate and spleen

It has been reported that some patients with CKD have an enlarged spleen, also called splenomegaly [258]. However, other structural changes in the spleen, such as calcifications, have not been described in CKD. As mentioned earlier, *Ahsg*^*−/−*^, *Enpp1* deficient, and *TNAP-Tg* mice develop calcifications in various soft tissues, which also includes the spleen [91, 95–97]. Furthermore, a transgenic mouse line overexpressing IL-5 shows calcifications in the spleen [259]. Similar to other tissues, *kl/kl* and *Fgf23*^*−/−*^ mice also develop atrophy in the spleen [181]. These studies suggest that hyperphosphatemia and inflammation might contribute to calcifications in the spleen. However, whether this also occurs in the context of CKD is currently unknown.

## Phosphate and thymus

CKD can cause thymus atrophy [260], which reduces T cell production and diversity, and might contribute to the impaired immune response in CKD patients [261]. Since *kl/kl* mice develop thymus atrophy [181, 262], hyperphosphatemia might contribute to this pathology. Calcifications in the CKD thymus have not been described. However, a histological analysis of aged mice revealed thymic dysfunction, including sustained fibrosis and calcification [263]. Calcifications in the thymus have been also described in *TNAP-Tg* mice [96].

## Phosphate and gonads

CKD is accompanied by reduced fertility as well as lower sexual function and activity in women and men [264, 265]. Ovarian reserve and reproductive hormones seem to be reduced in female CKD patients [265], and hypogonadism seems to be common in male patients with ESRD undergoing hemodialysis [266, 267]. While gonadal calcifications can occur in ovarian and testicular cancer [268], they have not been reported in CKD. Penile calcification have been reported in about 20% of patients with ESRD [269].

A potential association between high phosphate levels and testicular calcifications is indicated by studies in genetic diseases. For example, ultrasound and histopathological analyses of patients with familial tumoral calcinosis identified calcifications in the seminiferous tubules which was associated with decreased sperm production [270]. *Fgf23*^*−/−*^ mice with hyperphosphatemia develop calcifications in the testis and epididymis as well as spermatogenic arrest [271]. Furthermore, *kl/kl* and *Fgf23*^*−/−*^ mice show atrophy and impaired cell maturation in gonadal cells, and they develop hypogonadism and infertility [102, 106, 169, 170, 173, 181, 182]. However, a high-phosphate diet in healthy mice does not induce testicular calcifications [271]. Interestingly, PAM seems to associate with severe testicular microcalcifications [198, 207], suggesting that changes in local but not systemic phosphate levels might drive the calcification. Seminal fluid is rich in phosphate [67], which might contribute to the risk for calcifications. Reductions in inhibitors of calcification also seem to promote testicular calcifications. For example, CT analyses of *Ahsg*^*−/−*^ mice detected extensive calcification in the testis [97, 98], which might contribute to decreased breeding performance. Furthermore, testicular calcifications are observed in PXE patients with reduced PPi levels [272]. Testicular calcifications have been also described in other scenarios, such as in hypogonadal mice with deletion of the androgen receptor [273], in mice with deletion of *Slc9a3*, which encodes for a Na^+^/H^+^ exchanger [274], and in mice exposed to heat [275]. Overall, it appears that testicular calcifications contribute to impaired spermatogenesis and infertility, which needs further investigations. Whether testicular and ovarian calcifications occur in CKD and, if so, contribute to infertility is currently not known.

## Phosphate and the nervous system

CKD has various impacts on the central and peripheral nervous system, resulting in a spectrum of neurological complications, including cognitive impairments and motor abnormalities [276]. Elevated phosphate seems to affect the nervous system in different ways, as previously reviewed [277–279]. However, a causal interconnection between hyperphosphatemia and neurological complications in CKD has not been described to date. Nevertheless, calcifications in different brain areas have been reported in nephrectomized rats receiving a high-phosphate diet [250]. The finding that lowering serum phosphate levels in this model reduces brain calcifications suggests a causative involvement of phosphate. Neuronal calcifications and a potential contribution of elevated phosphate have been also identified in other mouse models. PiT-1 and PiT-2 are highly expressed in the brain [280–282], and mice with global *PiT-2* deletion develop basial ganglia calcification and calcification in brain arterioles [283]. In these mice, phosphate levels are elevated in the cerebrospinal fluid. *PiT-2* mutations are found in idiopathic basial ganglia calcification (IBGC; also called primary familial brain calcification/PFBC or Fahr’s disease), a rare genetic disease with calcification in basal ganglia, thalamus and cerebellum [284, 285]. In IBGC patients, mutations are also found in the xenotropic and polytropic retrovirus receptor 1 (XPR1) which mediates phosphate export from cells [286, 287]. It is possible that in IBGC brain calcifications are driven by local increases in extracellular phosphate levels, as also postulated for lung calcifications in PAM caused by *NaPi-2b* deletion. However, PiT-1 and PiT-2 seem to regulate the function of hippocampal neurons, and thereby learning and memory, independently of their phosphate transport activity [282], suggesting the existence of other pathomechanisms. Pathologic actions of hyperphosphatemia on the brain have been also suggested by studies in *kl/kl* mice, which develop cognition impairment [288], hearing disturbance [289], and motor neuron degeneration [290]. These phenotypes are also observed in mice lacking FGF23 [104]. Calcifications of the dura, which is the connective tissue membrane that forms the outermost layer of the meninges and protects the brain, have been reported in patients with familial tumoral calcinosis [117]. *Abcc6* and *Enpp1* deficiencies in mice or humans do not seem to be accompanied by severe brain calcifications or by neurological damage and cognitive impairments in general. However, histological alterations, including minor calcifications, have been described in the cerebral parenchyma of patients with PXE [291]. Besides rare genetic diseases, also CKD and hyperphosphatemia seem to cause brain calcifications, but larger clinical and experimental CKD studies are needed to strengthen our understanding. Overall, brain calcification is a common incidental finding in neuroimaging that is often associated with aging, trauma and infections, but often lacks a clear correlation with specific symptoms [292].

CKD also affects the eye, and corneal and conjunctival calcifications have been reported in dialysis patients [293–295]. Several other studies in humans [296], mice [297] and rats [298] reported similar patterns of calcification in the cornea in other contexts than CKD. A recent experimental study in mice receiving an adenine-rich diet combined with a high-phosphate diet for several weeks reported calcifications, calcium-phosphate depositions and elevated calcium content in the cornea [299]. Furthermore, these CKD mice develop calcified lesions in between the corneal epithelial and stromal layers in the eyes. Interestingly, this study also showed that treatment of cultured corneal epithelial cells with high phosphate induces osteogenic differentiation and calcification [299], suggesting that in CKD-associated corneal calcification direct effects of elevated phosphate on epithelial cells drive the calcification process. The potential contribution of high phosphate to calcifications in the eye is further supported by studies in animal models of genetic diseases with deficiencies in calcification inhibitors. For example, mice carrying a missense mutation for *Enpp1* exhibit calcification in the retina which worsens when magnesium content in the diet is reduced [93]. Similarly, *Abcc6*^*−/−*^ mice and rats develop calcifications in the retina, sclera, and conjunctiva of the eye [49, 84, 251, 300]. Histological alterations in the eye have been also described in patients with PXE and GACI [301, 302]. Furthermore, *Enpp1* deficiency in mice and in GACI patients is accompanied by hearing loss which might be attributable to calcifications [303, 304]. Moreover, calcifications in the eye have been reported in patients with tumoral calcinosis, including in the conjunctiva and in the area between the retina and capillaries [117].

## Conclusions

**Fig. 4:**
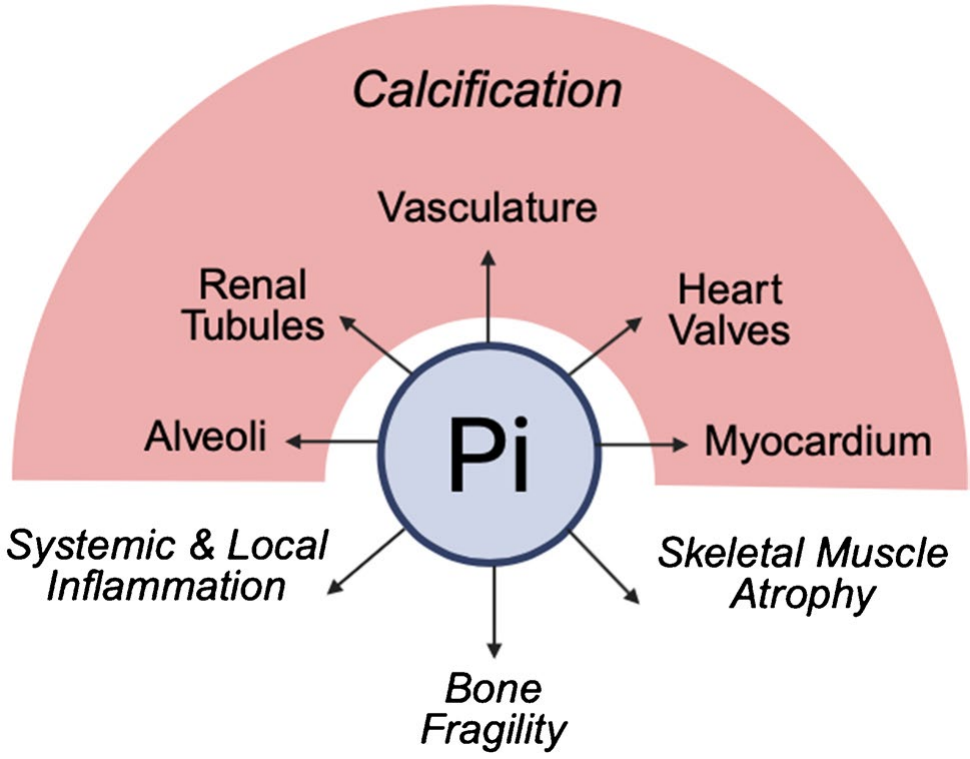
The pathologic actions of elevated phosphate in CKD. It is established that high concentrations of inorganic phosphate (Pi) cause vascular and valvular calcifications in patients with CKD. Calcifications might also occur within the myocardium, but this area needs further investigation. Similarly, CKD might be associated with alveolar calcification, which also needs to be studied in more detail. The impaired capacity to excrete Pi seems to cause an accumulation of Pi in the renal tubular lumen and the formation of calcium-Pi crystals, leading to calcifications in the kidney. While in CKD soft tissues calcify, the bone undergoes decalcification leading to a weakening of the bone structure, which seems to be also associated with high Pi levels. It is possible that elevated Pi also drives pathologic events other than calcifications. This includes the induction of inflammation, which could further support ectopic calcifications, as well as the reduction of protein mass in skeletal muscle fibers leading to muscle atrophy

It is plausible to assume that high phosphate levels can induce calcifications in other cell types than vascular smooth muscle cells and thereby cause tissue calcifications outside of the vasculature (Fig. 4). The prevalence of such extravascular calcifications in CKD and their appearance in relation to disease stages needs to be established. If it turns out that such ectopic extravascular calcifications are clinically relevant and associate with CKD morbidity and mortality, it will be important to better understand the precise pathomechanisms and cell types that are involved. Furthermore, it should be determined if these ectopic calcifications are reversible and treatable. To tackle these important questions, animal studies are inevitable. As a first step, existing animal models with CKD and hyperphosphatemia, which are known to develop vascular calcifications, should be evaluated for potential extravascular calcifications in different tissues. These studies could include histological stains to detect calcium-phosphate crystal depositions and the analysis of osteogenic gene programs. Elevated phosphate concentrations might have pathologic actions beyond the induction of ectopic calcifications (Fig. 4), which needs to be evaluated in a tissue-specific manner by using different experimental approaches.

Several factors can contribute to ectopic calcifications, which includes increases in local phosphate levels that might occur in the presence or absence of phosphate elevations in the circulation. Therefore, experimental studies should not only include the analysis of phosphate levels in serum, but also in tissues, using colorimetric or mass spectrometry approaches. Furthermore, the absence or dysfunction of the network that protects the organism from the formation and precipitation of calcium-phosphate crystals outside of bone, is a potent driver of ectopic calcifications. Ectopic calcifications might be most severe when these pathologic factors act together, such as in late stages of CKD, where phosphate concentrations are elevated and systemic levels of PPi, Mg and fetuin-A are low. It will be challenging to understand the precise molecular targets and pathways of elevated phosphate, since phosphate does not necessarily act on its own. However, even without a detailed understanding of the underlying pathomechanisms, it is worth to pursue therapeutic avenues that aim to lower systemic phosphate levels, which has been shown to have beneficial effects in various animal studies of CKD [7, 305]. Unfortunately, many of the promising experimental findings could not be translated into humans to date. Oral phosphate binders, which together with dietary phosphate restriction, are the primary treatment approaches to tackle hyperphosphatemia in dialysis patients, seem to have minimal efficacy in lowering serum phosphate levels and improving mineral metabolism and vascular health [306]. However, pharmacological approaches that tackle hyperphosphatemia by inhibiting phosphate uptake in the gut or in the kidney show more promising results in pre-clinical and clinical CKD studies [307–309]. Based on findings discussed here, future clinical studies should not only focus on measuring phosphate levels in the circulation but also in tissues, since tissue elevations might be the true culprit of hyperphosphatemia. However, it is obvious that it will be challenging to conduct such tissue analyses in humans. Furthermore, combining therapies that aim to lower systemic phosphate levels with treatments that increase the levels of calcification inhibitors, might be the most potent approach to protect from widespread vascular and extravascular calcifications [310].

## Data Availability

No datasets were generated or analysed during the current study.
